# Approximating the DCJ distance of balanced genomes in linear time

**DOI:** 10.1186/s13015-017-0095-y

**Published:** 2017-03-09

**Authors:** Diego P. Rubert, Pedro Feijão, Marília Dias Vieira Braga, Jens Stoye, Fábio Henrique Viduani Martinez

**Affiliations:** 1 Faculdade de Computação, Universidade Federal de Mato Grosso do Sul, Campo Grande, MS Brazil; 2 Faculty of Technology and Center for Biotechnology (CeBiTec), Bielefeld University, Bielefeld, Germany

**Keywords:** Double cut and join (DCJ), Genome rearrangements, Comparative genomics, Approximation algorithms

## Abstract

**Background:**

Rearrangements are large-scale mutations in genomes, responsible for complex changes and structural variations. Most rearrangements that modify the organization of a genome can be represented by the double cut and join (DCJ) operation. Given two balanced genomes, i.e., two genomes that have exactly the same number of occurrences of each gene in each genome, we are interested in the problem of computing the rearrangement distance between them, i.e., finding the minimum number of DCJ operations that transform one genome into the other. This problem is known to be NP-hard.

**Results:**

We propose a linear time approximation algorithm with approximation factor *O*(*k*) for the DCJ distance problem, where *k* is the maximum number of occurrences of any gene in the input genomes. Our algorithm works for linear and circular unichromosomal balanced genomes and uses as an intermediate step an *O*(*k*)-approximation for the minimum common string partition problem, which is closely related to the DCJ distance problem.

**Conclusions:**

Experiments on simulated data sets show that our approximation algorithm is very competitive both in efficiency and in quality of the solutions.

## Background

Large-scale mutations or rearrangements can produce complex changes and structural variations in genomes. They include inversions of chromosome segments (also called reversals), translocations of chromosome ends, fusions and fissions of chromosomes. All these rearrangements can be represented by the *double cut and join* (DCJ) operation [1], which basically consists of cutting a genome in two distinct positions (possibly in two distinct chromosomes) and joining the four resultant open ends in a different way.

A basic task in comparative genomics is to find the rearrangement distance between two given genomes, i.e., the minimum number of rearrangements that transform one genome into the other. For genomes without duplicate genes, there are linear time algorithms to compute the distance allowing only DCJ operations [2]. On the other hand, for genomes with duplicate genes, computing the rearrangement distance is NP-hard, even when the genomes have exactly the same number of occurrences of each gene in each genome (*balanced genomes*) and only DCJ operations are allowed [3, 4].

In this paper we design an approximation algorithm for computing the DCJ distance between two unichromosomal balanced genomes. The main step of our approximation algorithm is similar to approximating the NP-hard problem of computing the *Breakpoint Distance* (BD) in the presence of duplicate genes [5]. Let *k* be the maximum number of occurrences of any gene in the input genomes. With this parameter, BD has a 1.1037-approximation if $k = 2$ and a 4-approximation if $k = 3$ [6]. Otherwise, for general values of *k*, it has an *O*(*k*)-approximation [7, 8]. The latter result is based on a linear time approximation algorithm for the minimum common string partition (mcsp) problem [6] with approximation factor *O*(*k*) [9].

As we will show, the algorithm we developed to compute the DCJ distance of balanced genomes also has an approximation factor *O*(*k*) and linear running time. It works properly on inputs that are linear unichromosomal genomes. In addition, we describe how to extend it for circular unichromosomal genomes.

Experiments on simulated data sets show that our approximation algorithm is very competitive both in efficiency and in quality of the solutions.

A preliminary version of this paper appeared in the Proceedings of the 16th Workshop on Algorithms in Bioinformatics (WABI 2016) [10].

### Preliminaries

A *gene* *g* is an oriented DNA fragment that can be represented by the symbol *g* itself, if it has direct orientation, or by the symbol $\overline{g}$, if it has reverse orientation ($\overline{\overline{g}}=g$). A *chromosome* is a linear or a circular sequence of genes, and a *genome* is a set of chromosomes. Each one of the two ends of a linear chromosome is a *telomere*, represented by the symbol $\circ$.

Each chromosome in a genome can be represented by a sequence of genes that can be circular, if the chromosome is circular, or linear and flanked by the symbols $\circ$, if the chromosome is linear. Given a gene *g*, let $m_A(g)$ be the number of occurrences of *g* in a genome *A*. To refer to each occurrence of a gene *g* unambiguously, we number the occurrences of *g* from 1 to $m_A(g)$. When there exists at least one gene that occurs more than once in genome *A*, we say that *A* has *duplicate genes*.

In this work we consider only unichromosomal genomes, that are genomes composed of a single chromosome. Consider for instance the linear unichromosomal genome $A=(\circ \;c_1\;\overline{a}_1\;d_1\;b_1\;\overline{a}_2\;c_2\;\circ )$. In *A* we have one occurrence of genes *b* and *d* and two occurrences of genes *a* and *c*, that is, *A* has duplicate genes, and $m_A(a) = 2$, $m_A(b) = 1$, $m_A(c) = 2$ and $m_A(d) = 1$.

We use the notations $\mathcal {G}(A)$ and $\mathcal {G}^N(A)$, respectively, to refer to the set of (non-numbered) genes and to the set of numbered genes of a genome *A*. Considering again the genome *A* above, we have $\mathcal {G}(A) = \{a,b,c,d\}$ and $\mathcal {G}^N(A) = \{a_1,a_2,b_1,c_1,c_2,d_1\}$. Observe that the genomes $A'=(\circ \;c_2\;\overline{a}_1\;d_1\;b_1\;\overline{a}_2\;c_1\;\circ )$, $A''=(\circ \;c_1\;\overline{a}_2\;d_1\;b_1\;\overline{a}_1\;c_2\;\circ )$ and $A'''=(\circ \;c_2\;\overline{a}_2\;d_1\;b_1\;\overline{a}_1\;c_1\;\circ )$ are equivalent to $A=(\circ \;c_1\;\overline{a}_1\;d_1\;b_1\;\overline{a}_2\;c_2\;\circ )$. Given a genome *A*, possibly with duplicate genes, we denote by [*A*] the equivalence class of genomes that can be obtained from *A* by swapping indices between occurrences of the same gene.

### Balanced genomes

Let *A* and *B* be two unichromosomal genomes, possibly with duplicate genes. If they contain the same number of occurrences of each gene, i.e. $\mathcal {G}^N(A)=\mathcal {G}^N(B)$, we say that *A* and *B* are *balanced*. We can then simply denote by $\mathcal {G}=\mathcal {G}(A)=\mathcal {G}(B)$ the set of (non-numbered) genes and by $\mathcal {G}^N=\mathcal {G}^N(A)=\mathcal {G}^N(B)$ the set of numbered genes of *A* and *B*. For example, for balanced genomes $A = (\circ \;c_1\;\overline{a}_1\;d_1\;b_1\;c_2\;c_3\;\circ )$ and $B = (\circ \;a_1\;c_3\;\overline{c}_1\;\overline{b}_1\;d_1\;c_2\;\circ )$ we have $\mathcal {G} = \{a, b, c, d\}$ and $\mathcal {G}^N = \{a_1, b_1, c_1, c_2,c_3, d_1\}$.

### DCJ operations

Rearrangements can change the organization of a genome, i.e., the number of chromosomes in a genome or the order and the orientation of its genes. In general, such a rearrangement cuts a genome in two different positions, creating four open ends, and joins these open ends in a different way. It can be modeled by a *double-cut and join* (DCJ) operation [1]. Consider, for example, a DCJ applied to genome $(\circ \;c_1\;\overline{a}_1\;d_1\;b_1\;\overline{a}_2\;c_2\;\circ )$ that cuts before and after $\overline{a}_1\;d_1$, creating the segments $(\circ \;c_1\;\bullet )$, $(\bullet \;\overline{a}_1\;d_1\;\bullet )$ and $(\bullet \;b_1\;\overline{a}_2\;c_2\;\circ )$, where the symbol $\bullet$ represents the open ends. If we then join the first with the third and the second with the fourth open end, we obtain $(\circ \;c_1\;\overline{d}_1\;a_1\;b_1\;\overline{a}_2\;c_2\;\circ )$. This DCJ corresponds to the inversion of contiguous genes $\overline{a}_1\;d_1$. In general genomes, DCJ operations can also correspond to other rearrangements, such as translocations, fusions and fissions [1].

### DCJ distance and adjacency graph

Observe that the DCJ operation alone can only sort balanced genomes. We formally define the DCJ distance problem:

#### **Problem**

DCJ-distance(*A*, *B*): Given two balanced genomes *A* and *B*, compute their DCJ distance $d _\textsc {dcj} (A, B)$, i.e., the minimum number of DCJ operations required to transform *A* into $B'$, such that $B' \in [B]$.

Any sequence of $d _\textsc {dcj} (A, B)$ DCJ operations transforming *A* into $B' \in [B]$ is called an *optimal* sequence of DCJ operations.

Given two balanced genomes *A* and *B*, $d _\textsc {dcj} (A, B)$ can be computed with the help of the following concepts. First note that, since a gene *g* has an orientation, we can distinguish its two ends, also called its *extremities*, and denote them by $g^t$ (*tail*) and $g^h$ (*head*). An *adjacency* in a genome is an unordered pair of consecutive extremities in its chromosome (one of the two extremities can be a telomere). Thus, a genome *A* can also be defined as a set of adjacencies $adj (A)$ of its numbered genes. Given genome $A=\{(\circ \;c_1\;\overline{a}_1\;d_1\;b_1\;\overline{a}_2\;c_2\;\circ )\}$, for example, we have $adj (A) = \{\;\circ c_1^t\;,\;c_1^ha_1^h\;,\;a_1^td_1^t\;,\;d_1^hb_1^t\;,\;b_1^ha_2^h\;,\;a_2^tc_2^t\;,\;c_2^h\circ \;\}$.

Given two balanced genomes *A* and *B*, the *adjacency graph* $\textit{AG}(A, B)$ [2] is a bipartite multigraph such that each partition corresponds to the set of adjacencies of one of the two input genomes, and an edge connects the same gene extremities of adjacencies in both partitions, regardless of their index numbers. We say that the edge *represents* those extremities. If *A* and *B* are linear, each of the two telomeres of *A* must be connected by an edge to each of the two telomeres of *B*.

#### Without duplicate genes

First we consider the case when the genomes *A* and *B* contain no duplicate genes. If *A* and *B* are circular, there is a one-to-one correspondence between the set of edges in $\textit{AG}(A, B)$ and the set of gene extremities. In this case, all vertices have degree two and thus the adjacency graph is a collection of disjoint cycles. Here, problem DCJ-distance can easily be solved in linear time [1, 2] using the formula

$$\begin{aligned} d _\textsc {dcj} (A, B) = n - c, \end{aligned}$$

where $n = |adj (A)| = |adj (B)| = |\mathcal {G}|$ is the number of adjacencies or genes in any of the two genomes and *c* is the number of cycles in $\textit{AG}(A, B)$.

If *A* and *B* are linear, besides the edges connecting gene extremities, each telomere of *A* must be connected by an edge to each telomere of *B*. There is then an ambiguity concerning the vertices that contain a telomere, that have degree three. This means that we need to choose one of the two possible matchings of telomeres to obtain a graph in which all vertices have degree two, that is, a graph that is composed of cycles only. We must choose a matching that maximizes the number of cycles in the resulting $\textit{AG}(A, B)$. To accomplish this task, we just need to do a walk on the graph starting in one telomere of *A* until we find the next telomere in $\textit{AG}(A, B)$. If the second telomere is also in *A*, then we can pick any of the two possible matchings. In this case we have one big cycle covering all four vertices that contain a telomere. If the second telomere is in *B*, then we can pick the matching that connects these two telomeres (and consequently connects the other two telomeres, that were not covered by this walk). In this case we have two cycles covering the four vertices that contain a telomere. Once this matching is defined, problem DCJ-distance can again be solved in linear time [1] using the formula

$$\begin{aligned} d _\textsc {dcj} (A, B) = n - c, \end{aligned}$$

where $n = |adj (A)| = |adj (B)| = |\mathcal {G}| + 1$ is the number of adjacencies in any of the two genomes and *c* is the number of cycles in $\textit{AG}(A,B)$.

#### With duplicate genes

When genomes have duplicate genes, problem DCJ-distance becomes NP-hard [4]. In the same paper, the authors present an exact, exponential-time algorithm for its solution, phrased in form of an Integer Linear Program (ILP).

### An approach to compute the DCJ distance with duplicate genes

Observe that, in the presence of duplicate genes, the adjacency graph may contain vertices of degree larger than two. A *decomposition* of $\textit{AG}(A, B)$ is a collection of disjoint cycles covering all vertices of $\textit{AG}(A, B)$.

There can be multiple ways of selecting a decomposition of the adjacency graph. We need to find one that allows to match each occurrence of a gene in genome *A* with exactly one occurrence of the same gene in genome *B* and each telomere of *A* to one telomere of *B*. In order to build such a decomposition, we need the following definitions.

**Fig. 1:**
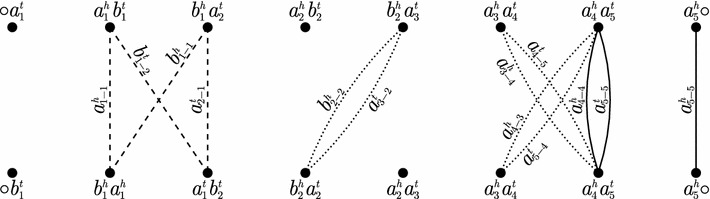
Examples of an inconsistent cycle (*dashed edges*) and an inconsistent set of cycles (*dotted edges*). The adjacency graph for $A = (\circ \; a_1 \; b_1 \; a_2 \; b_2 \; a_3 \; a_4 \; a_5 \; \circ )$ and $B = (\circ \; b_1 \; \overline{a}_1 \; b_2 \; a_2 \; a_3 \; a_4 \; a_5 \; \circ )$, with some edges omitted. For the sake of clarity, edges are labeled with extremities they represent. For example, an edge labeled $g^t_{i-j}$ represents extremities $g^t_i$ from *A* and $g^t_j$ from *B*

Let $g_i$ and $g_j$ be, respectively, occurrences of the same gene *g* in genomes *A* and *B*. The edge *e* that represents the connection of the head of $g_i$ to the head of $g_j$ and the edge *f* that represents the connection of the tail of $g_i$ to the tail of $g_j$ are called *siblings*. Two edges are *compatible* if they are siblings, if they represent the connection of extremities of distinct occurrences of the same gene, or if they represent the connection of extremities of distinct genes. Otherwise they are *incompatible*. A set of edges is *compatible* if it has no pair of incompatible edges. A cycle *C* of $\textit{AG}(A, B)$ is *consistent* if the set *E*(*C*) of edges of *C* is compatible. Note that, when constructing a decomposition, by choosing consistent cycles one may still select incompatible edges that occur in separate cycles (see the three dotted cycles of length 2 in Fig. 1). Thus, consistency cannot be taken into account in cycles separately.

A set of cycles $\{C_1, C_2, \ldots , C_k\}$ of $\textit{AG}(A, B)$ is *consistent* if and only if $E(C_1) \cup E(C_2) \cup \cdots \cup E(C_k)$ is compatible. A *consistent decomposition* *D* of $\textit{AG}(A, B)$ is a consistent set of disjoint cycles that cover all vertices in $\textit{AG}(A, B)$. Observe that in a consistent decomposition *D* we have only pairs of siblings, i.e., either an edge *e* and its sibling *f* are in *D* or both *e* and *f* are not in *D*. Thus, a consistent decomposition corresponds to a matching of occurrences of genes and telomeres in both genomes and allows us to compute the value

$$\begin{aligned} d_{D} = n-c_D, \end{aligned}$$

where $n = |adj (A)| = |adj (B)|$ and $c_D$ is the number of cycles in *D*. Observe that $n= |\mathcal {G}^N|$ if *A* and *B* are circular. If *A* and *B* are linear genomes, then $n= |\mathcal {G}^N| + 1$.

We can now compute the DCJ distance of two unichromosomal balanced genomes.

#### **Theorem 1**

*Given two unichromosomal balanced genomes A and B, the solution for the problem* DCJ-distance *is given by the following equation:*

$$\begin{aligned} d _\textsc {dcj} (A, B) = \min _{D \in \mathcal {D}}\{d_{D}\}, \end{aligned}$$

where $\mathcal {D}$ *is the set of all consistent decompositions of* $\textit{AG}(A, B)$.

#### *Proof*

Since a consistent decomposition allows to match duplicates in both genomes, clearly $d _\textsc {dcj} (A, B) \le \min _{D \in \mathcal {D}}\{ d_{D} \}$. Now, assume that $d _\textsc {dcj} (A, B) < \min _{D \in \mathcal {D}}\{ d_{D} \}$. By definition, this distance corresponds to an optimal rearrangement scenario from *A* to some $B' \in [B]$ and therefore implies a matching between the genes of *A* and the genes of $B'$. Furthermore, this matching gives rise to a consistent decomposition $D'$ of *AG*(*A*, *B*) such that $d_{D'} < \min _{D \in \mathcal {D}}\{ d_{D} \}$, which is a contradiction. $\square$

A consistent decomposition *D* such that $d_{D}=d _\textsc {dcj} (A, B)$ is said to be *optimal*.

Once a consistent decomposition *D* of the adjacency graph $\textit{AG}(A, B)$ is found, following [2] it is easy to derive in linear time a DCJ rearrangement scenario with $d_{D}$ DCJ operations transforming *A* into *B*. Moreover, an optimal consistent decomposition allows to find all optimal rearrangement scenarios [11].

## Results

Actually, all definitions and properties for the DCJ distance of balanced genomes presented from the beginning to here work properly for the general case, where genomes can be multichromosomal. However, as we will see in this section, to solve the DCJ distance problem we use an intermediate procedure whose inputs are strings. For this reason we restricted our inputs to *unichromosomal* genomes. Moreover, for the moment we will additionally consider only *linear* unichromosomal genomes, discussing later how to deal with *circular* unichromosomal genomes. The extension to multichromosomal genomes is left as an open problem.

### Approximating the DCJ distance by cycles of length 2

As mentioned above, given two linear unichromosomal balanced genomes *A* and *B*, we have to find a consistent decomposition of $\textit{AG}(A,B)$ to compute the DCJ distance according to Theorem 1. Recall that this is an NP-hard problem [4].

Given a consistent decomposition $D \in \mathcal {D}$ of the adjacency graph $\textit{AG}(A,B)$, we can see that

$$\begin{aligned} d_{D} = n - c_D = n - c_2 - c_>, \end{aligned}$$

where $n = |adj (A)|= |adj (B)|$, $c_2$ is the number of cycles of length 2, and $c_>$ is the number of cycles of length longer than 2 in *D*.

**Fig. 2:**
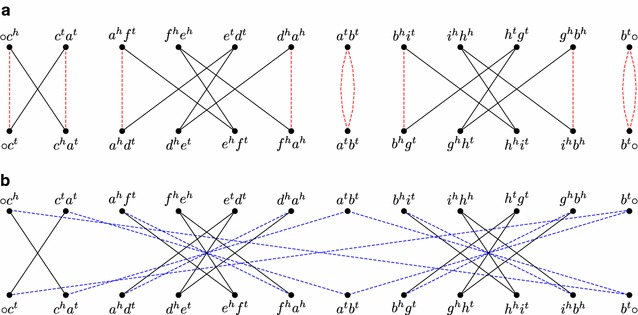
Two consistent decompositions for the same pair of genomes. The genomes (with gene indices omitted) are $A= (\circ \; \overline{c} \; a \; f_1 \; \overline{e} \; d \; \overline{a} \; b \; i \; \overline{h} \; g \; \overline{b} \; \circ )$ and $B= (\circ \; c \; a \; d \; e \; f \; \overline{a} \; b \; g \; h \; i \; \overline{b} \; \circ )$. Solid edges are in both decompositions. **a** A consistent decomposition $D'$ containing the maximum number of cycles of length 2, composed of 2 cycles of length 2, 1 cycle of length 4 and 2 cycles of length 8, resulting in $d_{D'} = 12 - 5 = 7$. **b** An optimal consistent decomposition $D^*$, composed of 6 cycles of length 4, resulting in $d_{D^*} = 12 - 6 = 6$

Building a consistent decomposition by maximizing $c_2$ as a first step is itself an NP-hard problem [12]. Furthermore, this strategy is not able to optimally solve the DCJ distance, as we can see in Fig. 2. Nevertheless, it allows us to approximate the DCJ distance:

#### **Lemma 2**

*A consistent decomposition* $D'$ *of* $\textit{AG}(A,B)$ *containing the maximum number of cycles of length 2 is a 2-approximation for the* DCJ-distance *problem.*

#### *Proof*

Let $c^*_2$ and $c^*_>$ be the number of cycles of length 2 and longer than 2, respectively, of an optimal consistent decomposition $D^*$ of $\textit{AG}(A,B)$. Let $c'_2$ and $c'_>$ be the numbers analogous to $c^*_2$ and $c^*_>$ with respect to the decomposition $D'$. It it easy to see that $c^*_2 + 2c^*_> \le n$, thus

1 $$\begin{aligned} 0&\le n - c^*_2 - 2c^*_> \nonumber \\ n- c^*_2&\le n - c^*_2 - 2c^*_> + n - c^*_2 \nonumber \\ n - c^*_2&\le 2(n - c^*_2 - c^*_>). \end{aligned}$$

Therefore, we have

2 $$\begin{aligned} \frac{d_{D'}}{d_{D^*}}&= \frac{n - c'_2 - c'_>}{n - c^*_2 - c^*_>}\nonumber \\&\le \frac{n - c^*_2 - c'_>}{n - c^*_2 - c^*_>} \end{aligned}$$

3 $$\begin{aligned}&\le \frac{n - c^*_2}{n - c^*_2 - c^*_>}\nonumber \\&\le \frac{2 (n - c^*_2 - c^*_>)}{n - c^*_2 - c^*_>}\end{aligned}$$

4 $$\begin{aligned}&= 2, \end{aligned}$$

where (2) holds since $c'_2 \ge c^*_2$, and (3) is true from (1). $\square$

### Minimum common string partition

The main result of this work relies on a restricted version of the minimum common string partition (mcsp) problem [6, 9], described briefly as follows.

Given a string *s*, a *partition* of *s* is a sequence $\mathcal {S} = [\mathcal {S}_1, \mathcal {S}_2, \ldots , \mathcal {S}_m]$ of substrings called *blocks* whose concatenation is *s*, i.e., $\mathcal {S}_1\mathcal {S}_2 \cdots \mathcal {S}_m = s$, and *m* is the *size* of $\mathcal {S}$.

Two strings *s* and *t* are *balanced* if any character has the same number of occurrences in *s* and in *t*, disregarding signs. Given two balanced strings *s* and *t* and partitions $\mathcal {S} = [\mathcal {S}_1, \ldots , \mathcal {S}_m]$ of *s* and $\mathcal {T} = [\mathcal {T}_1, \ldots , \mathcal {T}_m]$ of *t*, the pair $(\mathcal {S}, \mathcal {T})$ is a *common partition* of *s* and *t* if there exists a permutation *f* on $\{1, \ldots , m\}$ such that $\mathcal {S}_i = \mathcal {T}_{f(i)}$ for each $i = 1, \ldots , m$. The minimum common string partition problem (mcsp) is to find a common partition $(\mathcal {S},\mathcal {T})$ of two balanced strings *s* and *t* with minimum size.

We are interested in a restricted version of mcsp:

#### **Problem**

*k* -mcsp(*s*, *t*): Given two balanced strings *s* and *t* such that the number of occurrences of any character in *s* and *t* is bounded by *k*, find a common partition $(\mathcal {S},\mathcal {T})$ of *s* and *t* with minimum size.

Now let $\textit{occ}(A) = \max _{g \in \mathcal {G}(A)} \{m_A(g)\}$ be the maximum number of occurrences of any gene in a genome *A*. If two genomes *A* and *B* are balanced, we have $\textit{occ}(A) = \textit{occ}(B)$. For simplicity, in this case we use only $\textit{occ}$.

For a given linear unichromosomal genome *A*, let the *index-free* string $\widehat{A}$ be the gene sequence of the chromosome of *A* ignoring telomeres and gene indices. For example, for genome $A = (\circ \;c_1\;\overline{a}_1\;d_1\;b_1\;c_2\;c_3\;\circ )$, we have $\widehat{A}= c\overline{a}dbcc$.

### Finding consistent decompositions

In this section we present a linear time approximation algorithm Consistent-Decomposition, which receives two linear unichromosomal balanced genomes *A* and *B* with $\textit{occ}= k$ and returns a consistent decomposition of $\textit{AG}(A,B)$, which is an *O*(*k*)-approximation for the DCJ distance. The main steps of Consistent-Decomposition can be briefly described as follows.

First, from the input genomes *A* and *B*, we build their adjacency graph $\textit{AG}(A,B)$. We can then find a consistent decomposition by computing an approximation for *k* -mcsp($\widehat{A},\widehat{B}$), which gives an approximation for the number of breakpoints between genomes *A* and *B*. After that we remove the chosen cycles of length 2 from $\textit{AG}(A,B)$. Following, we iteratively collect arbitrary cycles of length longer than 2, removing them from the remaining graph after each iteration. Finally, we return the set of collected cycles as a consistent decomposition of $\textit{AG}(A,B)$. Pseudocode of Consistent-Decomposition is given in Algorithm 1. The individual steps are detailed in the following.
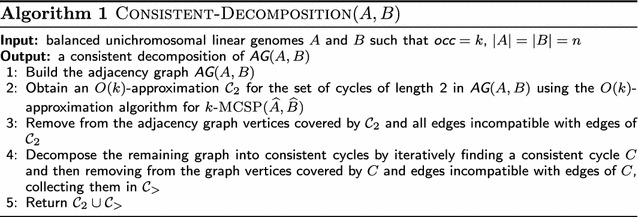


Step 1 of Consistent-Decomposition consists of building the adjacency graph of the given balanced genomes *A* and *B* as described previously. After that, Step 2 collects cycles of length 2 of $\textit{AG}(A,B)$ using an *O*(*k*)-approximation algorithm for *k*-mcsp($\widehat{A},\widehat{B}$) [9]. Step 3 removes from $\textit{AG}(A,B)$ vertices covered by cycles in $\mathcal {C}_2$ and edges incompatible with edges of cycles in $\mathcal {C}_2$.

Step 4 constructs the set $\mathcal {C}_>$ by decomposing the remaining graph into consistent cycles. Iteratively, it chooses a consistent cycle *C* and then removes from the remaining graph vertices covered by *C*. To find *C*, it can start with an empty path, choose some edge *e* from the remaining graph that extends the path and then remove from the remaining graph edges incompatible with *e* (just inspecting edges incident to vertices which are adjacent to *e* and to its sibling), repeating both edge selection and removal steps until the cycle is closed (it is easy to verify that this procedure will always close a consistent cycle). Hence the algorithm does not form an inconsistent cycle nor choose an inconsistent set of cycles. Further, this guarantees that for every edge in the decomposition, its sibling edge will also be in the decomposition. Note that $\mathcal {C}_>$ may contain cycles of length 2 not collected in $\mathcal {C}_2$.

A consistent decomposition of $\textit{AG}(A,B)$ is then the set $\mathcal {C}_2 \cup \mathcal {C}_>$, which is returned in Step 5.

To conclude this section, we present the following result which, together with the *O*(*k*) approximation algorithm for *k* -mcsp from [9], establishes an approximation factor for DCJ-distance.

#### **Theorem 3**

*Let A and B be linear unichromosomal balanced genomes such that* $\textit{occ}= k.$ *Let* $(\mathcal {A}, \mathcal {B})$ *be a common string partition with approximation factor* *O*(*k*)* for* *k* -mcsp($\widehat{A},\widehat{B}$). *A consistent decomposition D of* $\textit{AG}(A, B)$ *, containing cycles of length 2 reflecting preserved adjacencies in* $(\mathcal {A}, \mathcal {B})$ *, is an O*(*k*)*-approximation for the* DCJ-distance *problem.*

#### *Proof*

Let $c_2^*$ and $c_>^*$ be the number of cycles of length 2 and longer than 2, respectively, of an optimal consistent decomposition $D^*$ of $\textit{AG}(A, B)$. Let $\mathcal {C}_2$ be the set of cycles of length 2 reflecting preserved adjacencies in $(\mathcal {A}, \mathcal {B})$, and let $\mathcal {C}_>$ be an arbitrary consistent decomposition of cycles in $\textit{AG}(A, B) \setminus \mathcal {C}_2$. Let $D = \mathcal {C}_2 \cup \mathcal {C}_>$, a consistent decomposition, $c_2 = |\mathcal {C}_2|$, and $c_> = |\mathcal {C}_>|$. Since $(\mathcal {A}, \mathcal {B})$ is an *O*(*k*)-approximation of *k* -mscp, it follows that $n - c_2 \le \ell (n - c_2')$, where $\ell = O(k)$ and $c_2'$ is the number of cycles of length 2 in a consistent decomposition $D'$ with maximum number of cycles of length 2. Hence,

5 $$\begin{aligned} \frac{d_{D}}{d_{D^*}}&=\frac{n - c_2 - c_>}{n - c^*_2 - c_>^*}\nonumber \\&\le \frac{\ell \,(n - c'_2) - c_>}{n - c^*_2 - c_>^*}\nonumber \\&\le \frac{\ell \,(n - c'_2)}{n - c^*_2 - c_>^*}\nonumber \\&\le 2\ell \left( \frac{n - c_2' - c_>'}{n - c^*_2 - c_>^*}\right) \end{aligned}$$

6 $$\begin{aligned}&\le 4\ell , \end{aligned}$$

where (5) is analogous to (1) and (6) holds from (4), both in the proof of Lemma 2. $\square$

### Running time

Prior to addressing the running time of Consistent-Decomposition, we must consider one implicit but important step in the algorithm, which is to obtain the set $\mathcal {C}_2$ given the output of the *k* -mcsp approximation algorithm [9]. This algorithm takes as input $\widehat{A}$ and $\widehat{B}$ and outputs a common string partition $(\mathcal {A}, \mathcal {B})$.

Both $\mathcal {A}$ and $\mathcal {B}$ are composed of the same set of substrings, in different orders and possibly reversed, e.g., $\mathcal {A} = [\overline{ba}, a, ab]$ and $\mathcal {B} = [ab, ab, a]$ for index-free strings $\widehat{A}= \overline{ba}aab$ and $\widehat{B}= ababa$. Each substring of length $l > 1$ in $\mathcal {A}$ and $\mathcal {B}$ induces a sequence of $l - 1$ preserved adjacencies in $\widehat{A}$ and $\widehat{B}$. Then we just have to map each substring in $\mathcal {A}$ to the same substring in $\mathcal {B}$ (in case of multiple occurrences, we choose any of them). Considering $\mathcal {A}$ and $\mathcal {B}$ in the example above, *ab* and $\overline{ba}$ in $\mathcal {A}$ could be mapped to the first and second occurrences of *ab* in $\mathcal {B}$, respectively, since both *ab* and $\overline{ba}$ contain exactly the same preserved adjacency $a^hb^t$. We describe carefully in the next paragraphs the algorithm Substring-mapping (Algorithm 2) and how to use it to find such mapping while preserving the linear time complexity of Consistent-Decomposition.

The nontriviality of finding such mapping in linear time comes from the fact that alphabets of strings representing genomes are not constant size alphabets. They can and most likely will be of size *O*(*n*).

Before describing the algorithm, some observations and preprocessing must be addressed. We assume that the value *v*(*g*) of each symbol (gene family) *g* in the alphabet $\mathcal {G}$ is unique and in the range [1, *n*]. For reversed symbols we define $v(\overline{g}) = v(g) + n$, therefore their values will be in the range $[n+1,2n]$. Given different strings $s = s_1,\ldots ,s_\ell$ and $t = t_1,\ldots ,t_\ell$ of the same length $\ell$ such that *i* is the first position in which they differ, *s* is *lexicographically smaller* than *t* if $v(s_i) < v(t_i)$. (Note that $v(g) < v(\overline{g}$), therefore *g* comes before $\overline{g}$ lexicographically for any symbol *g*.)

**Fig. 3:**
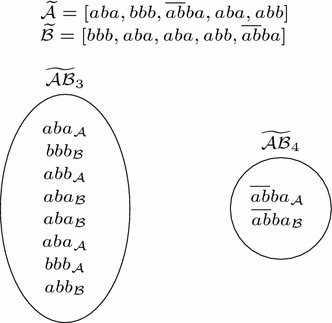
Example of the preprocessing step for the mapping of substrings. The subscript represents the origin of the string ($\mathcal {A}$ or $\mathcal {B}$), where $\mathcal {A} = [aba, bbb, \overline{ab}ba, \overline{aba}, abb]$ and $\mathcal {B} = [\overline{bbb}, aba, aba, abb, \overline{ab}ba]$


As preprocessing, we first create normalized versions $\widetilde{\mathcal {A}}$ of $\mathcal {A}$ and $\widetilde{\mathcal {B}}$ of $\mathcal {B}$, to ensure that for any substring *s*, only *s* or only its reverse $\overline{s}$ occurs in $\widetilde{\mathcal {A}} \cup \widetilde{\mathcal {B}}$. Therefore, for each string *s* in $\mathcal {A}$ (respectively $\mathcal {B}$), the normalized partition $\widetilde{\mathcal {A}}$ (respectively $\widetilde{\mathcal {B}}$) contains *s* itself, if *s* is lexicographically smaller than $\overline{s}$, and $\overline{s}$ otherwise. For instance, normalizing $\mathcal {A} = [\overline{ba}, a, ab]$ would change it to $\widetilde{\mathcal {A}} = [ab, a, ab]$. Also as a preprocessing step, given that we must find the same substrings in $\mathcal {A}$ and $\mathcal {B}$, it only makes sense to analyze substrings in both sets of the same length. Then, if there are substrings of multiple lengths in $\widetilde{\mathcal {A}}$ and $\widetilde{\mathcal {B}}$, in one pass through them (i.e. linear time) we can gather substrings of same length in buckets. Therefore, we define multisets $\widetilde{\mathcal {A}}_l = \{s\text { in }\widetilde{\mathcal {A}} : |s| = l\}$ (analogously $\widetilde{\mathcal {B}}_l$) and the generic bucket (multiset) $\widetilde{\mathcal {AB}}_l = \widetilde{\mathcal {A}}_l \cup \widetilde{\mathcal {B}}_l$ (also recording in some manner whether a string in $\widetilde{\mathcal {AB}}_l$ comes from $\mathcal {A}$ or $\mathcal {B}$), running the algorithm Substring-mapping for each bucket $\widetilde{\mathcal {AB}}_l$. See Fig. 3 for an example of this preprocessing step.

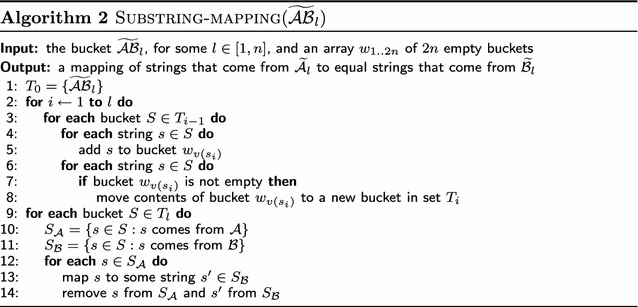


**Fig. 4:**
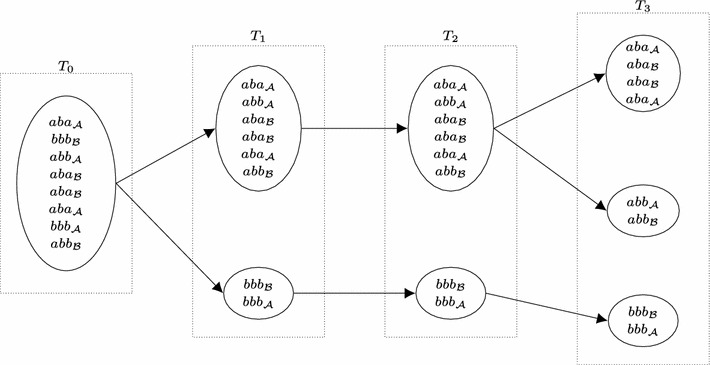
Example of the algorithm Substring-mapping for the bucket $\widetilde{\mathcal {AB}}_3$ of Fig. 3

The main idea of the algorithm Substring-mapping is, given a set of strings of length *l*, to obtain a set of buckets for some value of *i* (from 1 to *l*), each one containing strings which are found to be equal to the *i*th symbol, by splitting buckets for which strings are equal to the $(i-1)$st symbol. At the end, each bucket holds equal strings and we just have to map them taking into account their origin, $\mathcal {A}$ or $\mathcal {B}$. See an example in Fig. 4. Of course, instead of working with the substrings themselves we work just with references.

We shall demonstrate in the following lemma that this implicit mapping step can be performed in *O*(*n*) time:

#### **Lemma 4**

*The running time of *Substring-mapping *is proportional to the sum of lengths of strings in* $\widetilde{\mathcal {AB}}_l$,* for some* *l*.

#### *Proof*

Operations in lines 5, 7 and 8 can be done in constant time and are performed at most once per symbol of strings in $\widetilde{\mathcal {AB}}_l$. Operations in line 9 are performed *O*(1) times for each string in $\widetilde{\mathcal {AB}}_l$. Therefore, the total running time of Substring-mapping is $O(\sum _{s \in \widetilde{\mathcal {AB}}_l} |s|)$. $\square$

Since the buckets $\widetilde{\mathcal {AB}}_l$ are disjoint, we also have:

#### **Lemma 5**

*The set* $\mathcal {C}_2$ *can be obtained from the output of the* *k* -mcsp *approximation algorithm in* *O*(*n*)* time.*

#### *Proof*

Let $\widetilde{\mathcal {S}} = \{\widetilde{\mathcal {AB}}_l {:}$ there exists at least one substring of length *l* in $\widetilde{\mathcal {A}}$ (and therefore also in $\widetilde{\mathcal {B}}$)$\}$. To obtain $\mathcal {C}_2$, we must call Substring-mapping for each $\widetilde{\mathcal {AB}}_l \in \widetilde{\mathcal {S}}$, as noted before. The time complexity is the sum of time spent in all calls plus some extra preprocessing time. It is easy to see that $\widetilde{\mathcal {S}}$ can be obtained in one pass through $\widetilde{\mathcal {A}}$ and $\widetilde{\mathcal {B}}$, therefore in linear time. The array of buckets $w_{1.. 2n}$ can be defined in linear time once before calling Substring-mapping the first time and the buckets are empty at the end of each call. Finally, by Lemma 4 the running time of Substring-mapping for some $\widetilde{\mathcal {AB}}_l$ is linear in the sum of lengths of strings in $\widetilde{\mathcal {AB}}_l$, and the total sum of the lengths of strings in buckets $\widetilde{\mathcal {AB}}_l \in \widetilde{\mathcal {S}}$ is 2*n* (each substring of $\widetilde{\mathcal {A}}$ or $\widetilde{\mathcal {B}}$ appears once in exactly one $\widetilde{\mathcal {AB}}_l$). Hence, the total time complexity is *O*(*n*). $\square$

Having the running time of the implicit step of obtaining $\mathcal {C}_2$ by the output of the *k* -mcsp approximation algorithm, we can now analyze the complexity of Consistent-Decomposition.

#### **Theorem 6**

*Given linear unichromosomal balanced genomes* *A* *and* *B* *such that* $|A| = |B| = n$ *and* $\textit{occ}= k$,* the running time of algorithm* Consistent-Decomposition *is linear in the size of the genomes, i.e.,* *O*(*n*).

#### *Proof*

First, note that $\textit{AG}(A,B)$ is a bipartite graph composed of $2 (n + 1)$ vertices and at most $2kn + 4$ edges. This worst case occurs if there are $\lfloor {}n/k\rfloor$ gene families of size *k*, yielding $2k^2$ edges each ($k^2$ for the gene heads and $k^2$ for the gene tails), thus 2*kn* edges in total; plus 4 edges from the capping. Therefore, assuming *k* is a constant, $\textit{AG}(A,B)$ is of size *O*(*n*).

It is easy to see that Step 1 of Algorithm 1 has linear running time with respect to the size of $\textit{AG}(A,B)$, i.e. *O*(*n*). Computing the *k* -mcsp approximation [9] in Step 2 (with suffix trees for integer alphabets [13]) takes *O*(*n*) time. The same holds for the implicit step described above. The running time of Step 3 is *O*(*n*) since we have just to traverse vertices and edges of the remaining adjacency graph. Step 4 consists of collecting cycles arbitrarily and its running time is also linear, since we just have to walk in the remaining graph finding cycles and this can be done looking at each edge and each vertex at most *O*(1) times. The last step (Step 5) has running time *O*(1). Therefore, Consistent-Decomposition has running time *O*(*n*). $\square$

### Extending to circular unichromosomal genomes

**Fig. 5:**
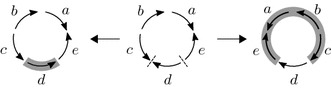
Example of two ways of performing a reversal in a circular chromosome (center). *Dashed lines* denote where cuts are made, *shaded regions* denote the reversed region. The two resulting chromosomes (sides) are the same

Meidanis et al. [14] showed that the problem of calculating the reversal distance for signed circular chromosomes without duplicate genes is essentially equivalent to the analogous problem for linear chromosomes (similar for transpositions in the unsigned case [15]). Therefore, any algorithm for the latter works for the former. The main idea is that each reversal on some region of a circular chromosome can be performed in two ways: reversing it directly or reversing everything else (Fig. 5).

In the following we show that similar ideas can also be applied to genomes with duplicate genes.

Let *A* and *B* be circular unichromosomal balanced genomes such that $\textit{occ}= k$. For some gene family *g*, there are in both *A* and *B* genes $g_1, g_2, \ldots , g_l$ with $l \le k$. Gene $g_1$ of *A* can be associated with *l* genes of *B*. We linearize *A* having $g_1$ with positive sign in the first position and linearize *B* *l* times, each one of them having one of the genes $g_1, g_2, \ldots , g_l$ with positive sign in the first position, associating it with $g_1$ (and assuming that both already are in the correct position). Next, we run Consistent-Decomposition on each one of the *l* linearizations, taking into account only the sequence of genes from position 2 to position *n*, keeping the best result. Thus, the running time of this strategy is $l \cdot O(n)$, that is, *O*(*n*) since $l \le k = \text {const}$.

#### **Corollary 7**

*For circular unichromosomal genomes* *A* *and* *B*,* the strategy of keeping the minimum output of* Consistent-Decomposition *for one linearization of* *A* *and* *l* *linearizations of* *B* *as described above leads to an* *O*(*k*)*-approximation for problem *DCJ-distance.

#### *Proof*

Let *d* be the DCJ distance between *A* and *B* and let $g_c$ be the copy of gene *g* in *B* associated to $g_1$ in *A* of the correct gene association to obtain *d*. One of the *l* linearizations of *B* associates $g_c$ in *B* with $g_1$ in *A*, approximating *d* with an *O*(*k*) factor by the Consistent-Decomposition algorithm. Clearly, the minimum output of all *l* linearizations will not be higher. $\square$

### Experimental results

We have implemented our approximation algorithm in C++, with the addition of a linear time greedy heuristic for the decomposition of cycles not induced by the *k* -mcsp approximation (available at https://git.facom.ufms.br/diego/k-dcj).

We compare our algorithm with Shao et al.’s ILP [4] (GREDU software package) on simulated datasets. Given two genomes, the ILP based experiments first build the adjacency graph, followed by capping of the telomeres, fixing some safe cycles of length two, and finally invoking an ILP solver to obtain an optimal solution with a time limit of 2 h. The experiments for both approaches were performed on an Intel i7 3.4GHz (4 cores) machine.

Following [4], we simulate artificial genomes with segmental duplications and DCJs. We uniformly select a position to start duplicating a segment of the genome and place the new copy to a new position. From a genome of *s* distinct genes, we generate an ancestor genome with 1.5*s* genes by randomly performing *s*/2*l* segmental duplications of length *l*, resulting in an average $k = 1.5$. Then we simulate two extant genomes from the ancestor by randomly performing *r* DCJs (reversals) independently. Thus, the simulated evolutionary distance between the two extant genomes is 2*r*. For each gene copy in the extant genomes we keep track of which gene copy in the ancestor it corresponds to, establishing the *reference bijection*, allowing us to compute the *true positive rate*, that is, for two genomes *A* and *B*, the rate of matchings of gene occurrences in *A* and *B* corresponding to the same gene occurrence in the ancestor genome.

**Fig. 6:**
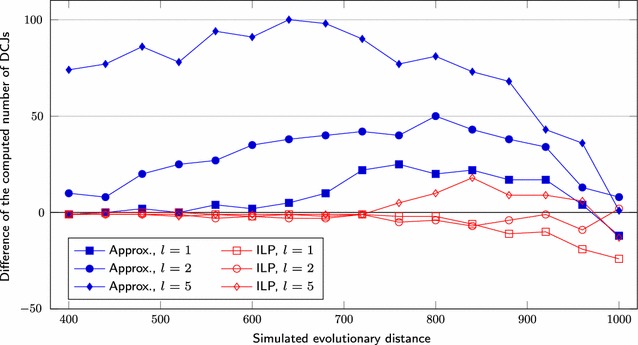
The computed number of DCJs vs. the simulated evolutionary distance for $s = 1000$

**Fig. 7:**
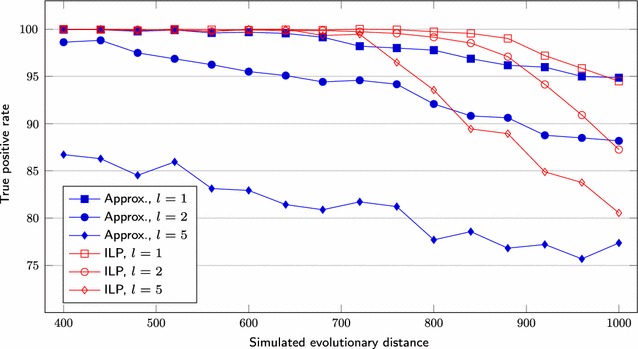
True positive rate for $s = 1000$

**Fig. 8:**
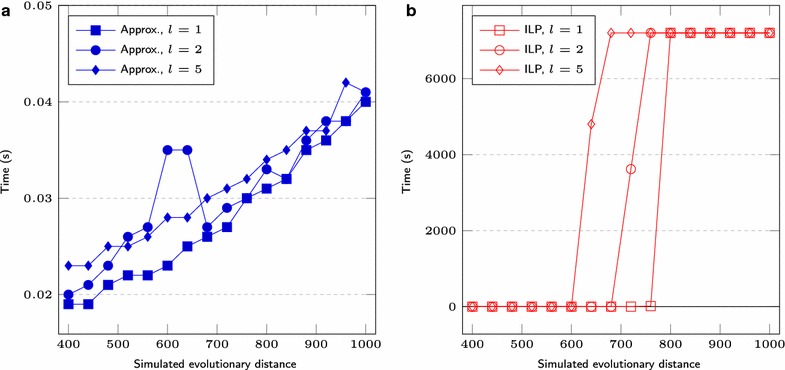
Execution time for $s = 1000$ of **a** approximation and **b** ILP based programs

**Fig. 9:**
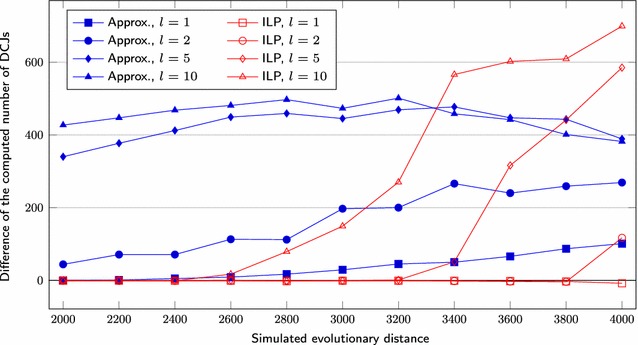
The computed number of DCJs vs. the simulated evolutionary distance for $s = 5000$

**Fig. 10:**
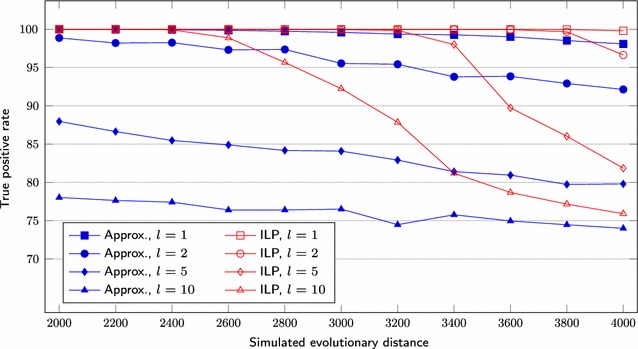
True positive rate for $s = 5000$

**Fig. 11:**
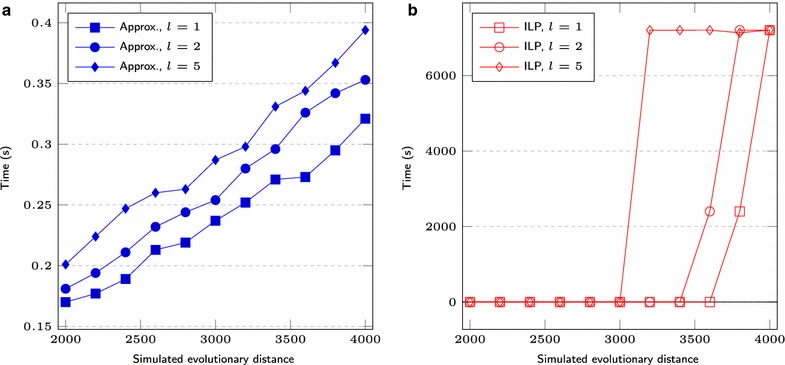
Execution time for $s = 5000$ of **a** approximation and **b** ILP based programs

We first set $s = 1000$, test three different lengths for segmental duplications ($l = 1, 2, 5$) and vary the *r* value over the range $200, 220, \ldots , 500$. We also simulate genomes having $s = 5000$, $l = 1, 2, 5, 10$ and *r* over the range $1000, 1100, \ldots , 2000$. Figures 6 and 9 show the average difference “*computed number of DCJs minus simulated evolutionary distance*”, taking as input three pairs of genomes for each combination of *l* and *r*, Figs. 7 and 10 show the *true positive rate*, while Figs. 8 and 11 show the average running times. Note that, although the DCJ distance is unknown, it is always less than or equal to the simulated evolutionary distance for these artificial genome pairs.

The difference of the number of DCJs (blue lines in Figs. 6,  9) calculated by our approximation algorithm remains very close to the simulated evolutionary distance for small values of *l*. Moreover, it remains roughly the same for the same value of *l* even for greater values of *r*. The values obtained by the ILP approach (red lines in Figs. 6,  9) are very close to those obtained by the approximation algorithm and to the simulated evolutionary distance from the simulations for $l \le 2$ and smaller values of *r*. However, beyond some point the DCJ distance calculated by the ILP gets even lower than the simulated evolutionary distance, showing the limitations of parsimony for larger distance ranges.

While the true positive rate is higher than 95% for most of datasets (Figs. 7,  10), the rate remains between 75 and 85% when $l \ge 5$ for the approximation approach and even for the ILP approach in some cases. For $s = 5000$ and $l \ge 5$, the computed number of DCJs increases while the true positive rate decreases significantly beyond some point for the ILP results. Notice that the approximation algorithm results for the same sets have small rates of increase or decrease, even for greater values of *r*.

The running time of our implementation of Consistent-Decomposition increases slowly from $\approx$0.03 s ($2r = 400$) to $\approx$0.08 s ($2r = 1000$) on average, when $s = 1000$, see Fig. 8a. The ILP approach takes $\approx$0.3 s for smaller values of *r* (where the preprocessing step fixes a considerable amount of cycles of length 2 in the adjacency graph), while always reaching the time limit of 2 h beyond some point, see Fig. 8b. A similar behavior is observed for $s = 5000$ (Fig. 11).

## Conclusion

In this paper, we have proposed a new approximation algorithm for the DCJ distance for genomes where each gene occurs the same number of times in each input genome and there exists at least one gene that occurs more than once in one of them. This so called DCJ distance with duplicates for balanced genomes problem is NP-hard [4]. Our algorithm works on input genomes where the amount of duplicates is bounded by *k*, the maximum number of duplicates of any gene in the input genomes. The approximation factor is *O*(*k*). Furthermore, our algorithm has linear running time in the size of the genomes. As experiments on simulated genomes have shown, our algorithm is very competitive both in efficiency and quality of the solutions, in comparison to an exact ILP solution.

Due to an intermediate step which approximates the minimum common string partition problem, our algorithm works properly only on unichromosomal genomes as input. A natural extension of this work is modifying it to work with multichromosomal genomes as well.
